# Process evaluation of an intervention to improve HIV treatment outcomes among children and adolescents

**DOI:** 10.5588/pha.22.0009

**Published:** 2022-09-21

**Authors:** M. Seguin, S. Dringus, S. Chiomvu, T. Apollo, E. Sibanda, V. Simms, S. Bernays, R. Chikodzore, N. Redzo, P. Mlilo, L. Ndlovu, P. Nzombe, B. Ncube, K. Kranzer, R. Abbas Ferrand, C. D. Chikwari

**Affiliations:** 1 Health Services Research and Policy, London School of Hygiene and Tropical Medicine, London, UK; 2 Global Health Department, London School of Hygiene and Tropical Medicine, London, UK; 3 Million Memory Project Zimbabwe, Bulawayo, Zimbabwe; 4 AIDS and TB Unit, Ministry of Health and Child Care, Harare, Zimbabwe; 5 Health Services Department, Bulawayo City Health, Bulawayo, Zimbabwe; 6 Biomedical Research and Training Institute, Harare, Zimbabwe; 7 International Statistics and Epidemiology Group, London School of Hygiene & Tropical Medicine, London, UK; 8 School of Public Health, University of Sydney, Sydney, NSW, Australia; 9 Ministry of Health and Child Care, Gwanda, Zimbabwe; 10 Clinical Research Department, London School of Hygiene & Tropical Medicine, London, UK; 11 Division of Infectious and Tropical Medicine, Medical Centre of the University of Munich, Munich, Germany

**Keywords:** antiretroviral therapy, adherence, community health workers

## Abstract

**SETTING:**

Children and adolescents with HIV encounter challenges in initiation and adherence to antiretroviral therapy (ART). A community-based support intervention of structured home visits, aimed at improving initiation, adherence and treatment, was delivered by community health workers (CHWs) to children and adolescents newly diagnosed with HIV.

**OBJECTIVES:**

To 1) describe intervention delivery, 2) explore CHW, caregiver and adolescents’ perceptions of the intervention, 3) identify barriers and facilitators to implementation, and 4) ascertain treatment outcomes at 12 months’ post-HIV diagnosis.

**DESIGN:**

We drew upon: 1) semi-structured interviews (*n* = 22) with 5 adolescents, 11 caregivers and 6 CHWs, 2) 28 CHW field manuals, and 3) quantitative data for study participants (demographic information and HIV clinical outcomes).

**RESULTS:**

Forty-one children received at least a part of the intervention. Of those whose viral load was tested, 26 (*n* = 32, 81.3%) were virally suppressed. Interviewees felt that the intervention supported ART adherence and strengthened mental health. Facilitators to intervention delivery were convenience and rapport between CHWs and families. Stigma, challenges in locating participants and inadequate resources for CHWs were barriers.

**CONCLUSION:**

This intervention was helpful in supporting HIV treatment adherence among adolescents and children. Facilitators and barriers may be useful in developing future interventions.

## Abstract (fr)

**CONTEXTE:**

Les enfants et les adolescents séropositifs rencontrent des difficultés dans l’initiation et l’adhésion à la thérapie antirétrovirale (TAR). Des agents de santé communautaires (CHW) ont mis en place une intervention de soutien communautaire sous forme de visites structurées à domicile visant à améliorer l’initiation, l’adhésion et le traitement, auprès d’enfants et d’adolescents nouvellement diagnostiqués séropositifs.

**OBJECTIFS:**

1) Décrire la mise en œuvre de l’intervention, 2) explorer les perceptions de l’intervention par les CHW, les soignants et les adolescents, 3) identifier les obstacles et les facilitateurs de la mise en œuvre, et 4) vérifier les résultats du traitement 12 mois après le diagnostic du VIH.

**METHODES:**

Nous nous sommes appuyés sur 1) des entretiens semi-structurés (*n* = 22) avec 5 adolescents, 11 soignants et 6 CHW, 2) 28 manuels de terrain des CHW, et 3) des données quantitatives sur les participants à l’étude (informations démographiques et résultats cliniques du VIH).

**RÉSULTATS:**

Quarante et un enfants ont reçu au moins une partie de l’intervention. Parmi ceux dont la charge virale a été testée, 26 (*n* = 32 ; 81,3%) étaient sous suppression virale. Les personnes interrogées ont estimé que l’intervention soutenait l’adhésion au TAR et renforçait la santé mentale. Les facilitateurs de la mise en œuvre de l’intervention étaient la commodité et les rapports entre les CHW et les familles. La stigmatisation, les difficultés à trouver des participants et les ressources inadéquates pour les CHW étaient des obstacles.

**CONCLUSION:**

Cette intervention a été utile pour soutenir l’adhésion au traitement du VIH chez les adolescents et les enfants. Les facilitateurs et les obstacles peuvent être utiles pour développer de futures interventions.

Adherence to antiretroviral therapy (ART) is critical to achieving viral suppression and optimal clinical outcomes in HIV care.^1^ Adherence to ART among adolescents is lower than among adults,^2^,^3^ resulting in poorer rates of viral suppression,^4^,^5^ and disproportionally high mortality rates.^1^ Interventions to increase linkage to care, ART initiation and adherence among this group are required.

Home-based support visits delivered by community health workers (CHWs) to children living with HIV and their caregivers positively impact ART adherence.^6^–^8^ The Zimbabwe Study for Enhancing Testing and Improving Treatment of HIV in Children (ZENITH) trial, which consisted of 15 CHW visits over 72 weeks to households of older children (6–15 years) with HIV in an urban setting in Zimbabwe improved attendance at medical appointments and viral suppression.^9^,^10^ However, ZENITH was an efficacy trial and the number of visits required considerable extra resources. The Bridging the Gap in HIV Testing and Care for Children in Zimbabwe (B-GAP) Project was thus conducted to test how this type of intervention performs in ‘real-life’ settings led by routine health providers without extra resources.

The B-GAP Project evaluated the provision of index-linked HIV testing for children and adolescents aged 2–18 years in routine health services, combined with support visits by CHWs for those who tested HIV-positive in urban (Bulawayo City) and rural (Mangwe District) settings.^11^ Caregivers of children newly diagnosed with HIV, or previously diagnosed but not linked to care, were offered an abbreviated version of the ZENITH intervention consisting of five home visits, followed by two optional visits delivered over 7 months to support initiation and adherence to ART, in addition to standard HIV clinical care (Table 1). Visits were delivered by pre-existing CHWs trained by B-GAP Project staff. CHWs were paid a small stipend for each visit. They kept a manual to record visit dates, whether objectives were met, participant progress and challenges delivering the visits. Two-monthly meetings were conducted between the B-GAP coordinator and CHWs to monitor progress, identify challenges and provide minimal support.

**TABLE 1:** Description of visits

Visit name	When?	Objectives
Initial visit	Within 1 month of diagnosis (all age groups: primary caregiver and child present)	Make initial contact with client Describe the home visit intervention Complete client details Introduce Hero Book Plan date for next visit (to occur within 2 weeks)
Planning for successful treatment	Within 1.5 months of HIV diagnosis (all age groups: primary caregiver and child present)	Answering questions regarding HIV arising from the clinic appointment Complete family mapping Identify strengths and resources available to the client Discussion of treatment experience to date Development of Personal Treatment Plan Continue Hero Book
Reviewing treatment plan and plan for disclosure	3 months following diagnosis (young children: primary caregiver and child present; older children, just child present)	Discussion of personal treatment plan to date Discussion of side effects and management strategies Discussion of barriers to adherence and strategies to overcome these Assessment of disclosure, and plan for disclosure Linkage to locally available support services, if needed Continue Hero Book
Disclosing and planning for the future	6 months following HIV diagnosis (young children: primary caregiver and child present; older children, just child present)	Discussion of personal treatment plan to date Discussion of side effects and management strategies Discussion of barriers to adherence and strategies to overcome these Follow-up on disclosure to child/others Linkage to locally available support services, if needed Preparing for hand-over and exit of programme Continue Hero Book
Handover and saying goodbye	7 months following diagnosis (all age groups: primary caregiver and child present)	Complete client details Complete “Hero in Me” in Hero Book Carry out hand-over of programme to client Say goodbye
Additional support	Optional: only if the family requires additional support	Review personal treatment plan Follow-up on any issues that arise Review: referrals, disclosure to the child and others, support from household members Discussion around long-term maintenance of treatment Address client concerns
Additional support	Optional: only if the family requires additional support	Review personal treatment plan Follow-up on any issues arising Review: referrals, disclosure to the child and others, support from household members Discussion about long-term maintenance of treatment Address client concerns

The aims of this mixed-methods process evaluation are to describe intervention delivery, explore caregivers’, adolescents’ and CHWs’ perceptions of the intervention, identify barriers and facilitators to implementation and ascertain treatment outcomes at 12 months’ post-HIV diagnosis.

## STUDY POPULATION, DESIGN AND METHODS

### Evaluation methods

‘Process evaluations’ typically examined implementation, mechanism of impact and context. ‘Implementation’ covered fidelity (intervention quality) and dose (quantity of what is delivered). ‘Mechanism of impact’ was understood in terms of how participants responded to, and interacted with, complex interventions.^12^ ‘Context’ referred to the social environment within which the intervention was delivered, and involved the exploration of how external factors influenced the delivery and functioning of interventions.^13^ Our process evaluation reports details of the intervention delivered (including dose), and examines mechanisms of impact and context by exploring perceptions of the intervention, including barriers and facilitators, held by CHWs, adolescents and caregivers. Our process evaluation drew on three data sources: 1) semi-structured interviews with CHWs, caregivers and adolescents (16–19 years) who received the intervention; 2) CHW field manuals; and 3) linkage to care and HIV viral load assessed at 12-months’ post-diagnosis.

Caregiver and adolescent interviewees were purposively recruited to achieve a heterogenous sample with respect to gender, age and location (rural and urban). Research staff invited participant caregivers, adolescents (aged ⩾16 years) and CHWs to take part in semi-structured interviews. Face-to-face recorded interviews were conducted in Shona, Ndebele or English in December 2019 by two research assistants (PN and BN) trained in qualitative data collection. Interview recordings were transcribed and translated (as needed) to produce an English transcript.

We followed a critical realist approach^14^ to capture the external reality affecting the intervention, as well as interviewee perceptions of this reality. Qualitative data from the CHW manuals and interview transcripts were coded deductively in Nvivo (QSR International, Melbourne, VIC, Australia). The coding tree covered perceptions of the intervention, as well as barriers and facilitators to its success by interviewee type (caregiver, adolescent, CHW). Quantitative data on outcomes were analysed using STATA v15 (StataCorp, College Station, TX, USA). Categorical variables were summarised as counts and percentages, and continuous variables as medians and interquartile ranges (IQRs). Viral suppression was defined as a viral load of <1000 copies/ml at 12 months’ post-diagnosis.

Ethical approval was obtained from the Medical Research Council of Zimbabwe, Harare (MRCZ/A/2167), the Institutional Review Board of the Biomedical Research and Training Institute, Harare, Zimbabwe (AP138/2017), and the London School of Hygiene & Tropical Medicine Ethics Committee, London, UK (12263-3). Written informed consent was obtained from all interviewees. The legal age for consent is 16 years in Zimbabwe. Adolescents aged 13–18 years signed an assent paragraph on the same consent form as adult participants.

## RESULTS

### Intervention delivery

A total of 50 children diagnosed with HIV using index-linked testing from February to December 2018 were eligible for this community-based support intervention. An additional six children diagnosed at satellite sites were also offered the intervention. The median age of the 56 children (40 and 16 from urban and rural settings, respectively) was 11 years (IQR 7.5–15); 36 (64.3%) were female.

Table 2 below shows the characteristics of the children eligible and receiving the intervention. Of the 56 eligible children, 41 (73.2%) started the intervention. Of the 15 who did not start the intervention, the majority (*n* = 12) were lost to follow-up. The reasons for not receiving the intervention were available for five. Of these 5, CHWs were unable to contact 2 caregivers due to incorrect phone numbers, 2 caregivers refused involvement when contacted by the CHWs and 1 child moved out of the study district. Results below are based on quantitative data for 41 children who started the intervention and 28 field manuals that were returned by CHWs (Table 2).

**TABLE 2:** Characteristics of children living with HIV who were eligible for and who received the intervention

Characteristic		Eligible for intervention (*n* = 56) *n* (%)	Received intervention (*n* = 41) *n* (%)*
Location	Urban	40 (71.4)	25 (61.0)
	Rural	16 (28.6)	16 (39.0)
Age, years	2–12	32 (59.3)	19 (50.0
	13–18	22 (40.7)	19 (50.0)
	Missing	2	3
Sex	Female	36 (64.3)	24 (58.5)
	Male	18 (32.1)	17 (41.5)
	Missing	2	0
Number of visits received	0	15 (26.8)	—
	1–4	14 (25.0)	14 (34.1)
	5–7	27 (48.2)	27 (65.9)
Registered with clinic	Yes	43 (76.8)	39 (95.1)
	No	3 (5.4)	2 (4.9)
	Unknown	10 (17.9)	0 (0)
Initiated on ART	Yes	43 (76.8)	39 (95.1)
	No/unknown	13 (23.2)	2 (4.9)
Ever had CD4 count	Yes	24 (42.8)	16 (39.0)
	No	14 (25.0)	13 (31.7)
	Unknown	28 (32.1)	12 (29.3)
Viral load test conducted^†^	Yes	32 (57.1)	27 (65.9)
	No	24 (42.9)	14 (34.2)
Viral load <1000 copies/ml	Yes	26 (81.1)	20 (74.1)
	No	6 (18.8)	7 (25.9)
	Not done	24	14

* Percentages do not always add to 100 due to missing data and rounding.

† Includes tests conducted during and after intervention.

ART = antiretroviral therapy.

Most children (27/41, 65.8%) received five visits. Of these, 19 received one or two additional support visits. Of those who received five visits, the time between the first and fifth visit ranged from 104 to 448 days, with a median of 185 days (6 months). The length of visits of the 146 visits recorded in the 28 CHW field manuals ranged from 15 to 308 min, with a median duration of 58 min. All objectives per visit (Table 3) were met for 95 of the 146 visits (65.0%), as recorded by the CHWs. Objectives related to introducing and working on the Hero Book (spanning several visits), the family mapping exercise in Visit 2, and assessment of and plan for disclosure in Visit 3 were met the least often.

**TABLE 3:** Characteristics of interview participants

Pseudonym	Type	Location, setting	Sex	Age range of interviewee	Age of child (sex)	Relationship to child	Length (min)	Number of attendees in interview
Hlengiwe	Adolescent	Bulawayo, home	Female	16–19	—	—	42	1
Lindiwe	Adolescent	Bulawayo, home	Female	16–19	—	—	45	1
Ayanda	Adolescent	Bulawayo, home	Male	16–19	—	—	37	2
Dumi	Adolescent	Bulawayo, home	Male	16–19	—	—	31	1
Sthabile	Adolescent	Mangwe, clinic	Female	16–19	—	—	31	1
Bandile	Caregiver	Bulawayo, clinic	Female	21–64	10 (F)	Aunt	37	1
Thokozile	Caregiver	Bulawayo, home	Female	21–64	12 (F)	Mother	28	3
Andile	Caregiver	Bulawayo, home	Female	21–64	5 (M)	Mother	47	2
Gugulethu	Caregiver	Bulawayo, home	Female	21–64	17 (M)	Grandmother to Ayanda	45	∼3
Lungile	Caregiver	Bulawayo, home	Female	21–64	18 (F)	Mother to Hlengiwe	46	2
Thando	Caregiver	Bulawayo, home	Female	21–64	8 (F)	Grandmother	44	∼3
Nomusa	Caregiver	Mangwe, clinic	Female	Unknown	13 (M)	Mother	40	∼3
Noxolo	Caregiver	Mangwe, clinic	Female	21–64	12 (M)	Sister	31	2
Sibongile	Caregiver	Mangwe, clinic	Female	21–64	11 (F)	Grandmother	37	1
Sibusisiwe	Caregiver	Mangwe, clinic	Female	21–64	8 (F)	Mother	44	2
Thandeka	Caregiver	Mangwe, clinic	Female	21–64	11 (F)	Mother	46	1
Sandile	CHW	Bulawayo, office	Male	21–48	—	—	17	1
Buhle	CHW	Bulawayo, office	Male	21–48	—	—	51	1
Sipho	CHW	Bulawayo, office	Male	21–48	—	—	41	1
Zodwa	CHW	Bulawayo, office	Female	21–48	—	—	39	1
Ntombizodwa	CHW	Mangwe, clinic	Female	21–48	—	—	53	1
Nokuphiwa	CHW	Mangwe, clinic	Female	21–48	—	—	31	2

F = female; M = male; CHW = community health worker.

### Perceptions of the intervention

The 22 interviewees consisted of five adolescents (aged 16–19 years), 11 caregivers (aged 21–64 years) and six CHWs (aged 21–48 years). There were two caregiver-adolescent pairs in the sample. Although an even gender split was achieved for CHW and adolescent participants, caregiver participants were exclusively female. Table 3 shows the characteristics of interviewees. All names are pseudonyms.

Most caregivers, adolescents and CHWs viewed the intervention as helpful in supporting ART commencement and adherence. Thokozile noted,

It’s heart-breaking to have a child who is always sick, but with the help of the health workers it became better. [caregiver]

Lindiwe explained:

I should take my medication every day and that I am not supposed to skip because if I stop, I might end up getting sick or getting other diseases. [adolescent]

Specific guidance on medication dosage and scheduling were noted by several caregivers as helpful information, as well as the implications of non-adherence. Andile stated,

I have been taught a lot of things concerning my son’s medication and what should be done in order for him to lead a healthy life. I have also been taught on the dangers of not giving him his medication. [caregiver]

Caregivers and CHWs felt that the intervention had helped children and adolescents in accepting their status, viewed as a necessary precursor to treatment commencement and adherence. Thokozile remarked,

I could not force her to take her medication because she still did not believe her status. […] I think the visits assisted me a lot because if it was not for them, I am sure she was not going to take her medication you see, and she would still be sick. [caregiver]

When asked how the intervention could be improved, the addition of food and/or monetary support to attend school or meet other needs were frequently mentioned by caregivers and adolescents. Lungile suggested:

You can provide us with food to enable us to take the tablets. We can’t really take this medication on an empty stomach. It exacerbates the hunger and sometimes makes you feel dizzy. [caregiver]

Several caregivers and adolescents felt that the end of the home visits was abrupt and would have appreciated the visits to continue. Thandeka reflected,

…they left a void in our lives and our children miss their good teachings and counseling guidance. [caregiver]

Lindiwe added,

I think [the visits] were too few. I wish we could continue with the visits so that we could have more discussions. [adolescent]

Caregivers, adolescents and CHWs reported mental health benefits for adolescents through participation in B-GAP. Lungile explained that the programme had given her daughter a more positive outlook, stating,

I think we could be going through a lot psychologically if they had not come. [caregiver]

CHWs noted that their clients’ self-esteem increased due to the intervention. Zodwa recalled,

Her self- esteem was low when I started vising her. […] [A]t first she felt like her life has ended but I just told her no this is not the end of life; you can even have more years to come. Being HIV-positive does not end one’s life. So now she is someone who has got self-esteem, she now knows where she came from and where she wants to go, and she even has the goals for the future. [CHW]

Some caregivers reported their *own* mental health had improved due to the intervention. Thandeka recalled that her assigned CHW consoled her and assisted her to cope with her 11-year-old daughter’s diagnosis, stating,

They gave me their shoulder to lean on during time of difficulty.

### Facilitators and barriers to intervention delivery

#### Facilitators

Caregivers and adolescents found the visit format beneficial. Bandile suggested that home visits reached those caregivers who were reluctant to seek assistance:

…some parents are ashamed to go out there and seek information on HIV, so this project helps a lot. [caregiver]

Sthabile appreciated the additional time available during CHW home visits vs. clinic appointments:

[T]he nurses at the clinic will be busy attending other patients whereas the community health workers […] have enough time for our discussions. [adolescent]

Several caregivers noted that CHWs took a flexible approach in scheduling visits, which allowed other activities such as chores and schooling to be accommodated.

The high level of rapport between CHWs, caregivers and adolescents facilitated intervention delivery. Sibusisiwe recalled that her 8-year-old daughter loved the visits and looked forward to them:

[I]t was a good relationship anchored on her teaching us a lot of life things. I would receive her knowing fully that she has come to assist us. [caregiver]

These sentiments were echoed by the adolescents. Dumi’s CWH made him feel less alone by revealing that he was also living with HIV:

I became free to relate to him and I always looked forward to his visits. [adolescent]

#### Barriers

Stigma interfered with intervention delivery, as it affected where and when visits could occur, how freely intervention recipients could communicate during visits and the degree to which caregivers and children engaged with the material. Sipho, a male CHW, recounted that one caregiver felt uncomfortable talking about her son’s HIV diagnosis due to concerns that neighbours may overhear the discussion. To address this, Sipho posed as the child’s tutor and developed code words with the caregiver for sensitive terms (such as HIV and ART) to prevent neighbours discovering the boy’s status.

CHWs noted that some topics were difficult for clients to discuss due to stigma. The session on family mapping required CHWs to establish the HIV status of household members, who were often reluctant to disclose this. Zodwa said:

I came across with this client whereby the father I asked him if he is [HIV+] since the wife and the daughter were HIV-positive. He told me that he wasn’t ok, his HIV status was positive, but I could tell that he did not want to tell me. [CHW]

Some children and adolescents refused to engage with CHWs due to denial. For example, Thokozile’s 12-year-old daughter initially refused to take part in the visits. She recounts,

My child was still in denial and she did not want to get involved. They would call and ask if they could come but I always came up with excuses because my child failed to believe that she was positive, and she refused to take her medication. […] I would sometimes tell them that I am not around. [caregiver]

This quote also illustrates how Thokozile managed her daughter’s reluctance to take part by telling CHWs that they were unavailable. This behaviour was reflected more widely, with CHWs commenting that some caregivers were evasive when they attempted to arrange visits.

### HIV care outcomes

At 12 months’ post-diagnosis, 30 (*n* = 56, 53.6%) children were found and interviewed by the study team to ascertain their treatment outcomes. Of the remaining 26 children, 4 (7.1%) caregivers, but not the children were found and interviewed; 12 (21.4%) were lost to follow-up and no records were found; only their clinical records were found in case of 5 children (8.9%); and the remaining 5 children moved out of the study districts. Of these five, medical records were found for four children (7.1%) (Figure).

**FIGURE:**
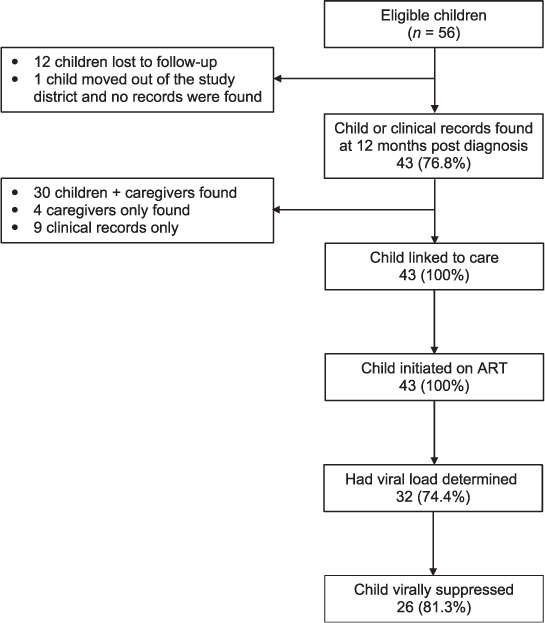
Child treatment outcomes at 12 months.

Of the 43 children whose records were available, all were linked to care (registered with a clinic) and initiated ART. Viral load tests or records of viral load test were found for 32/43 (74.4%) children who had registered for care; among these, 26 (81.2%) were virally supressed (<1000 copies/mL) (Figure). There were no reported deaths, although these cannot be excluded among those lost to follow-up. Of the 43 children with outcome data, 35 (81.3%) had received at least one home visit from a CHW.

## DISCUSSION

Nearly a quarter of children and adolescents were not linked to care and were lost to follow-up before they could be offered the intervention. The need for interventions supporting children and adolescents to link to care is clear.

Most of the children and adolescents who were linked to care received at least part of the intervention. Over 65% of children who started the intervention received more than five core home visits. CHWs, caregivers and adolescents felt that the visits should continue beyond the intervention period, demonstrating intervention acceptability. Both providers and recipients perceived that the intervention was beneficial in supporting children’s mental and physical health. Improvements in mental health may be a mechanism through which these visits may support treatment adherence, as several studies have highlighted the effect of poor mental health on ART adherence.^15^–^17^ Provision of support to caregivers of children and adolescents living with HIV is particularly important, as wellbeing of carers can have a significant impact on the care and outcomes of the people they are responsible for.^18^,^19^

Stigma was a significant barrier to intervention delivery, as it affected where and when visits could occur, how freely intervention clients could communicate during visits, and the degree to which clients engaged in the material covered during visits. This was also noted in the ZENITH trial, where some participants resisted home visits and were unreceptive to key messages.^20^ Effective interventions to reduce stigma may increase the acceptability of interventions improving linkage to care.

Among children who started the intervention and have available records, 81% had received a CHW visit and 80% were virally suppressed at 12 months. Viral load suppression of 80% was the target stipulated by the Zimbabwe Ministry of Health and Child Care for children accessing this intervention. While this does fall short of the UNAIDS “90-90-90” targets, it is higher than levels of viral suppression in this age group reported by other studies.^21^–^23^ However, it is important to note that 14 of the 41 children did not have a viral load measure available. As suggested elsewhere, this may indicate the need for CHW support visits over a longer period of time.^24^

The economic pressure caregivers were under may have hindered effective delivery of the intervention. It is critical that support programmes consider the context in which children with HIV live, and combine psychosocial support with economic support, which may improve HIV treatment outcomes, as well as overall wellbeing of the child and the household.^25^,^26^

A strength of this study was that the CHW intervention was delivered by cadres who were already part of the Zimbabwe healthcare system. The research team employed a ‘hands-off’ approach, providing minimal supervision to CHWs. This model demonstrates the feasibility of replication and potential scalability without adding resources to the healthcare system.

We acknowledge several limitations. Participants’ family members were occasionally present during interviews, which may have introduced desirability bias. Despite our efforts to select a gender-balanced sample, caregivers were exclusively female. This likely reflects the reality, as women (more than men) take on care-giving responsibilities.^27^ The degree to which objectives were met during visits were self-reported by the CHWs delivering the intervention, which may have biased the data.

Caregivers, adolescents and CHWs viewed this home visit intervention as helpful in supporting HIV treatment initiation and adherence among adolescents and children. Observed facilitators and barriers to impact may be useful in developing and supporting future ART adherence interventions for children and adolescents living with HIV in other lower and middle-income countries.
